# Visual Field Analysis: A reliable method to score left and right eye use using automated tracking

**DOI:** 10.3758/s13428-021-01702-6

**Published:** 2021-10-08

**Authors:** Mathilde Josserand, Orsola Rosa-Salva, Elisabetta Versace, Bastien S. Lemaire

**Affiliations:** 1 Center for Mind/Brain Sciences, University of Trento, Corso Bettini 31, 38068 Rovereto, TN Italy; 2 Laboratoire Dynamique Du Langage UMR 5596, Université Lumière Lyon 2, 14 avenue Berthelot, 69363 Lyon Cedex 07, France; 3 School of Biological and Chemical Sciences, Queen Mary University of London, London, E1 4NS UK; 4 Alan Turing Institute, London, NW1 2DB UK

**Keywords:** Automated tracking, Lateralization, DeepLabCut, Computational methods, Behavioural scoring

## Abstract

Brain and behavioural asymmetries have been documented in various taxa. Many of these asymmetries involve preferential left and right eye use. However, measuring eye use through manual frame-by-frame analyses from video recordings is laborious and may lead to biases. Recent progress in technology has allowed the development of accurate tracking techniques for measuring animal behaviour. Amongst these techniques, DeepLabCut, a Python-based tracking toolbox using transfer learning with deep neural networks, offers the possibility to track different body parts with unprecedented accuracy. Exploiting the potentialities of DeepLabCut, we developed Visual Field Analysis, an additional open-source application for extracting eye use data. To our knowledge, this is the first application that can automatically quantify left–right preferences in eye use. Here we test the performance of our application in measuring preferential eye use in young domestic chicks. The comparison with manual scoring methods revealed a near perfect correlation in the measures of eye use obtained by Visual Field Analysis. With our application, eye use can be analysed reliably, objectively and at a fine scale in different experimental paradigms.

## Introduction

Accurate quantification of animal behaviour is crucial to understanding its underlying mechanisms. Historically, behavioural measurements were collected manually. However, with technological progress, automated data collection and analyses have expanded (Anderson & Perona, 2014), making behavioural analyses more precise, reliable and effortless for the experimenter (Lemaire et al., 2021; Versace et al., 2020a, b; Wood & Wood, 2019). Thus, computational ethology constitutes a promising research avenue, especially for research on laterality (Vallortigara, 2021).

Automated data collection may allow finer and more objective behavioural analyses than the ones provided by manual coding. However, many of the currently available tracking techniques and software can be complex to use and even inaccurate in some experimental conditions (such as in poor and changing illumination, low contrast, etc.). The open-source toolbox DeepLabCut copes with these limitations (Mathis et al., 2018; Nath et al., 2019). DeepLabCut exploits deep learning techniques to track animals’ movements with unprecedented accuracy, without the need to apply any marker on the body of the animal (Labuguen et al., 2019; Mundorf et al., 2020; Worley et al., 2019; Wu et al., 2019), opening a new range of possibilities for measuring animal behaviour such as behavioural asymmetry data or preferential eye use. While different markerless software can track animals’ body parts (e.g. EthoVision XT, ANY-maze), we focus on DeepLabCut because it is open-source, accurate, does not require a pre-specified experimental setting and is widespread in behavioural research.

It is now clear that structural and functional asymmetries, once believed to be unique to humans, are widespread among vertebrates (Halpern et al., 2005; Rogers, 2015; Rogers et al., 2013; Vallortigara & Versace, 2017; Versace & Vallortigara, 2015) and invertebrates (Frasnelli, 2013; Frasnelli et al., 2012; Rogers, 2015). The study of sensory and perceptual asymmetries is a powerful tool for understanding functional lateralization, especially in animals with laterally placed eyes. For instance, in non-mammalian models, researchers can take advantage of anatomical features causing most of the information coming from each eye system to be processed by the contralateral brain hemisphere (Vallortigara & Versace, 2017). For example, birds have an almost complete decussation of the fibres at the optic chiasma (Cowan et al., 1961) and limited connections between the hemispheres due to a lack of corpus callosum (Andrew, 1991; Mihrshahi, 2006). Therefore, the information entering the left eye is mainly processed by the right hemisphere. With such anatomical structure, the preferential use of one eye most likely reflects the hemisphere in action. To date, temporary occlusion of one eye has been the main method used for behavioural investigation of eye asymmetries (Andrew, 1991; Chiandetti, 2017; Chiandetti et al., 2014; Chiandetti & Vallortigara, 2019; Güntürkün, 1985; Vallortigara, 1992; Vallortigara et al., 1999). However, studying spontaneous eye use without monocular occlusions is important to shed light on the lateralization of naturalistic behaviours. Indeed, a considerable amount of evidence has shown that animals actively use one or the other visual hemifield depending on the task (De Santi et al., 2001; Güntürkün & Kesch, 1987; Prior et al., 2004; Rogers, 2014; Santi et al., 2002; Schnell et al., 2018; Sovrano et al., 1999; Tommasi et al., 2000; Vallortigara et al., 1996) and/or motivational/emotional state (Andrew, 1983; Bisazza et al., 1998; De Boyer Des Roches et al., 2008; Larose et al., 2006).

A widely used method for testing preferences in eye use is the frame-by-frame analyses of video recordings (Fagot et al., 1997; Rogers, 2019). A drawback of this procedure is that it is tedious and may originate potential errors and biases (Anderson & Perona, 2014). Moreover, in animals with two foveae (or a ramped fovea) like birds, it is necessary to distinguish the use of frontal and lateral visual fields, making manual coding of these data even more complicated (Lemaire et al., 2019; Vallortigara et al., 2001). To address these issues, we developed an application for the automatic recording of eye use preferences for comparative neuroethological research. This application, named Visual Field Analysis, is based on DeepLabCut tracking (Nath et al., 2019) and enables eye use scoring as well as other behavioural measurements (see Josserand & Lemaire, 2020 for more details).

Measuring eye use is particularly relevant for species that have laterally placed eyes and therefore use their frontal and lateral visual fields differently (e.g., among birds, domestic chicks and king penguins, Vallortigara et al., 2001; Lemaire et al., 2019). The current study aims to experimentally validate the main function of Visual Field Analysis: scoring preferential eye use in animals. To do so, we assessed the accuracy of Visual Field Analysis in scoring preferential eye use of domestic chicks (*Gallus gallus*)*,* while looking at an unfamiliar stimulus, in comparison with traditional manual scoring (an approach that has been used, for instance, in Dharmaretnam & Andrew, 1994; De Santi et al., 2001; Vallortigara et al., 2001; Rogers et al., 2004; Dadda & Bisazza, 2016; Schnell et al., 2016). Note that our application can also be used to measure other variables such as the level of locomotor activity (a measurement used in open field or runway tests, e.g., Gallup & Suarez, 1980; Gould et al., 2009; Ogura & Matsushima, 2011), and the time spent by an animal in different areas of a test arena (a measure widely used in recognition, generalization and spontaneous preference tests, which measure animals’ preferences between two or more stimuli, Wood, 2013; Rosa-Salva et al., 2016; Versace et al., 2016, Versace et al. 2020). For further details on the functioning and current limitations of this application, please see the full protocol published by Josserand and Lemaire (2020).

## Methods

### Subjects

The experimental procedures were approved by the Ethical Committee of the University of Trento and licenced by the Italian Health Ministry (permit number 53/2020). We used 10 chicks of undetermined sex (strain Ross 308). The eggs were obtained from a commercial hatchery (Azienda Agricola Crescenti) and incubated at the University of Trento under controlled conditions (37.7 °C and 40% humidity). Three days before hatching, we moved the eggs into a hatching chamber (37.7 °C and 60% humidity). Soon after hatching, the chicks were housed together in a rectangular cage (150 × 80 × 40 cm) in standard environmental conditions (30 °C and homogeneous illumination, adjusted to follow a natural day/night cycle) and in groups of a maximum of 40 individuals. Food (chick starter crumbs) and water were available ad libitum. The animals were maintained in these conditions for three days, until the test was performed. After the test, all animals were donated to local farmers.

### Test

**Fig. 1:**
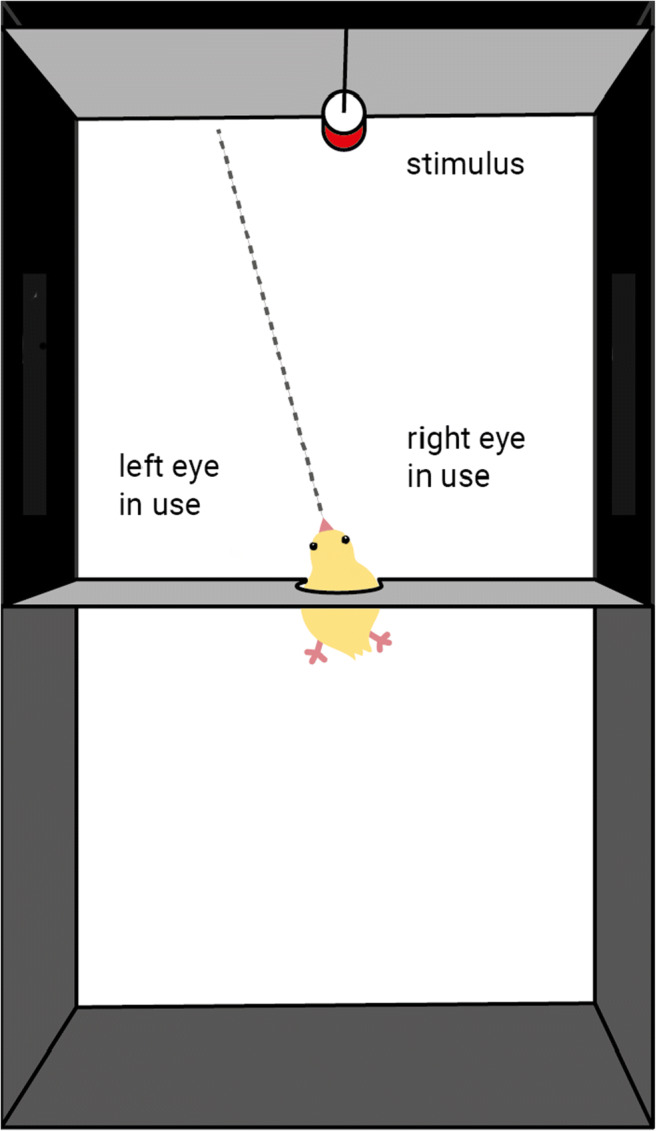
Schematic representation of the testing condition for Experiment 1 (top view). In chicks, eyes are placed laterally on the head’s side, creating wide monocular visual fields often used for visual exploration of objects, and a small binocular overlap (see also Fig. 3). Due to the structure of the visual field of chicks (and other birds with laterally placed eyes), the right monocular visual field is projected on the right eye and the left visual field is projected on the left eye

The test took place the third day post-hatching. Each chick was moved into an adjacent room and placed in a smaller experimental cage (45 × 20 × 30 cm) to begin the pretest habituation phase, which usually lasted about 30 minutes. The cage had a round opening (4 cm), and during the habituation phase the animal could pass its head through it at will, to inspect an additional empty compartment (20 × 20 × 30 cm, see Fig. 1). Young chicks tend to spontaneously perform this behaviour when given the opportunity. Once a subject was confidently passing its head through the opening, the proper test phase began, and a red cylinder (5 cm high, 2 cm in diameter) was added in the additional compartment (20 cm away). The subject’s head was then gently placed through the round opening by the experimenter and the animal was manually kept in this position for 30 seconds (Fig. 1). The behaviour of each animal was recorded with an overhead camera (GoPro Hero 5, 1290 × 720, ~ 25–30 fps) for 30 seconds. Each animal was tested only once.

### Data acquisition using Visual Field Analysis

#### Data acquisition

**Fig. 2:**
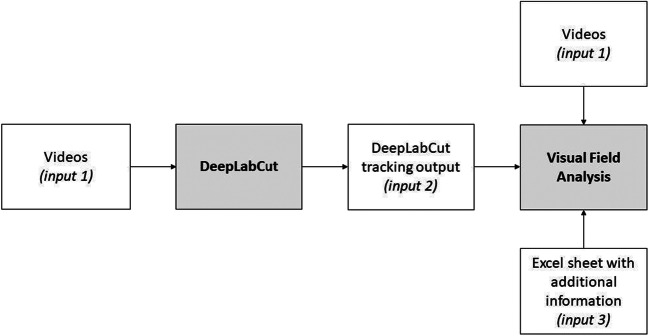
Diagram showing the inputs required to run Visual Field Analysis

To perform data acquisition, Visual Field Analysis requires three main inputs for each analysed subject (Fig. 2). The first input is the video recording of the animal’s behaviour. The second input is a file containing information about the tracking (*x*, *y* coordinates) of specific body parts (output file provided by DeepLabCut). Visual Field Analysis focuses on three points located on the head: the closest points to the left eye, the right eye and the top of the head. For the current experiment, the positions of these three points were manually labelled on 100 frames so that DeepLabCut could accurately generalize each point of interest on all video recordings. The third input corresponds to a spreadsheet where the experimenter manually enters specific information about the observed animal. Further information is provided in our protocol (Josserand & Lemaire, 2020).

**Fig. 3:**
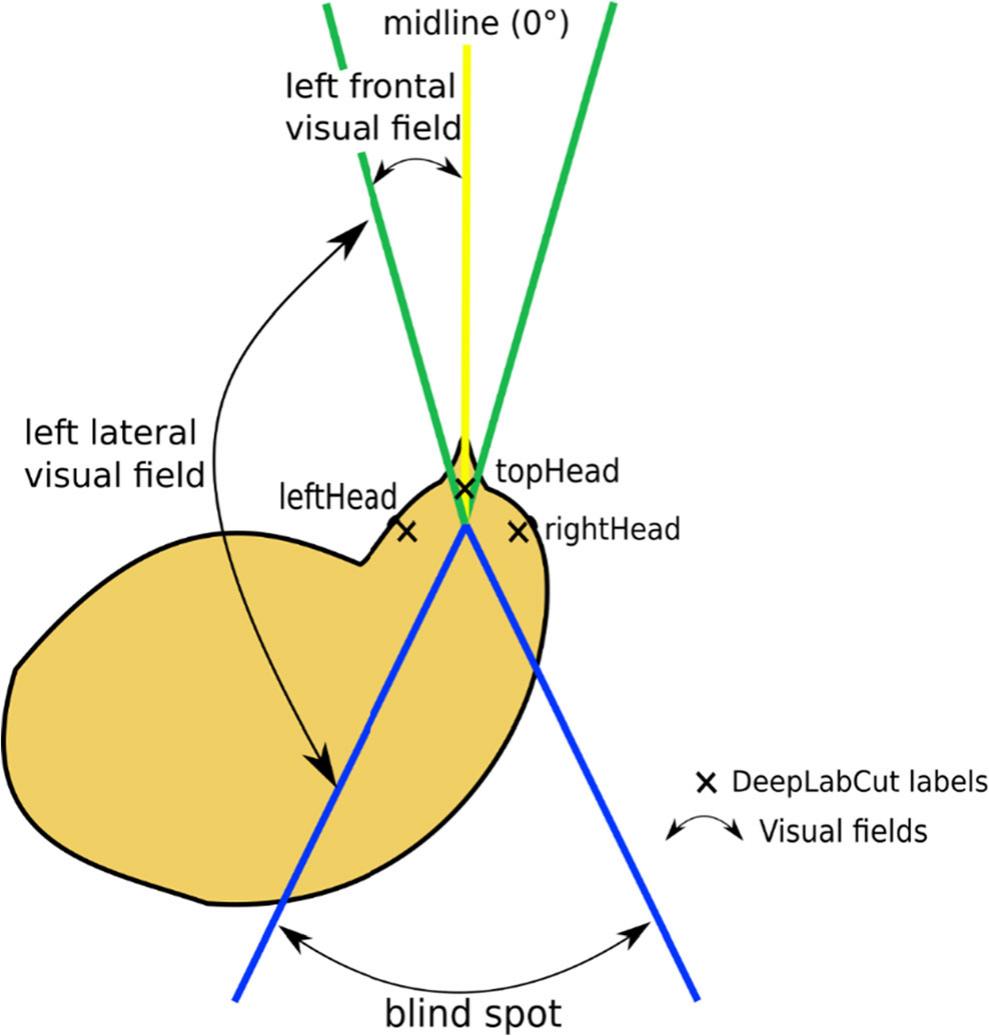
Schematic representation of a chick and its visual fields defined for the experiments. The yellow midline separates the left visual field from the right visual field. The green lines show the borders of the frontal vision (from the midline, 15° on each side), and the blue lines show the blind spot of the chick. Each angle can be manually chosen in the Visual Field Analysis program

To proceed with data acquisition, the extent of the frontal and lateral visual fields for the species under investigation must also be defined. In the current study, we subdivided the visual field as follows: two frontal visual fields (each 15° wide from the midline, Fig. 3), two lateral visual fields (each 135° wide starting from the frontal visual field line, Fig. 3) and the blind visual field (30° wide starting from the lateral visual field line, Fig. 3).

**Fig. 4:**
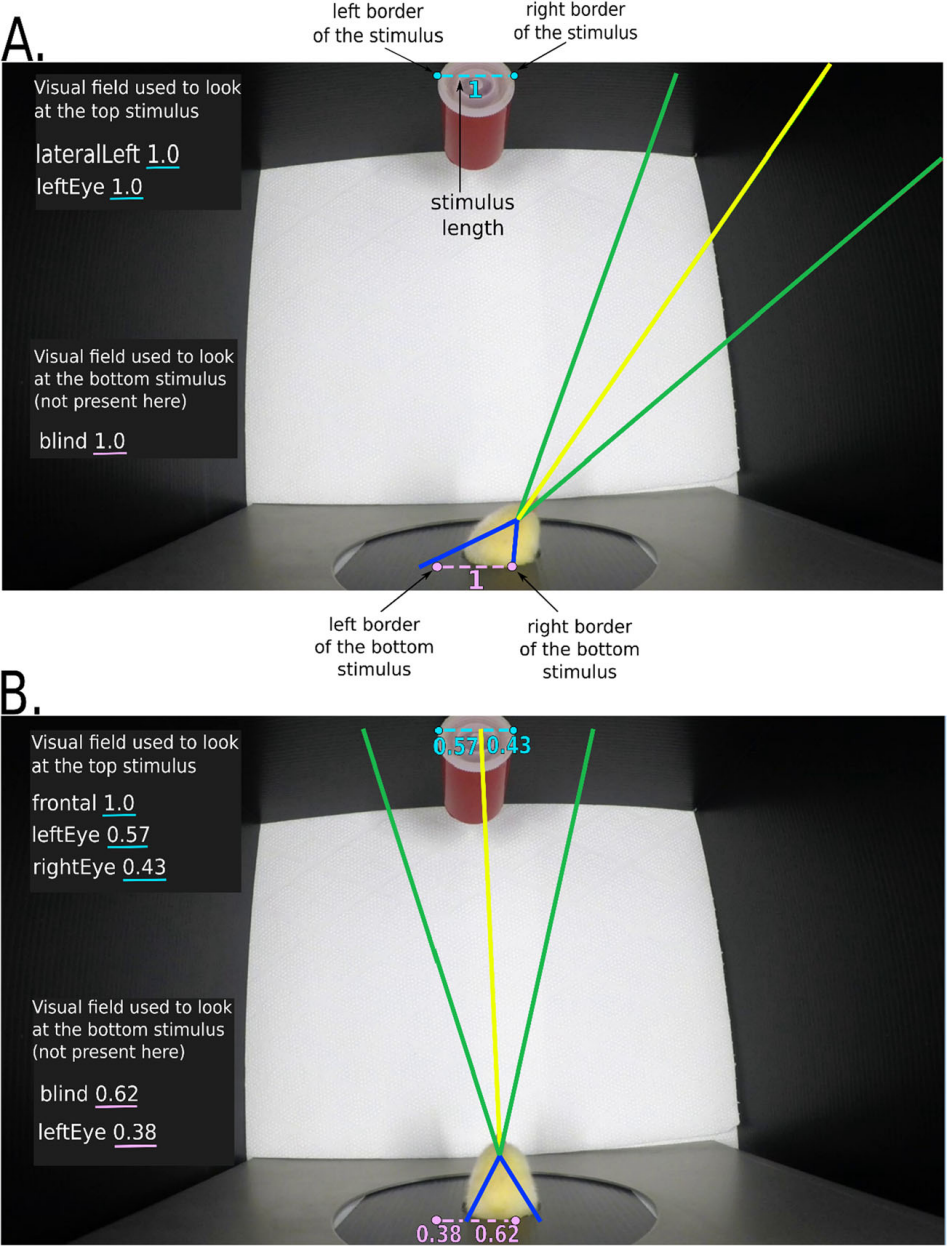
Visualization of the projection lines defining each region of the visual field. The yellow line indicates the midline and delimitates each hemifield, providing the nasal margin of the frontal visual fields. The green lines delimitate the frontal visual fields from the lateral visual fields. The blue lines delimitate the lateral visual fields from the blind spot. In these pictures, referring to the setup of the current experiment, the visual field used to look at the stimulus is shown, which can be compared to the information reported on the left side of the pictures (within the dark rectangles on the top and bottom left corners of the images). Since our application allows the recording of visual field use for up to two simultaneously presented objects, in these images we can see two grey rectangles (reporting information on the eye use for each of the two objects). In the current example, where only one stimulus was present, the relevant information is presented in the grey rectangle in the upper part of the image (referring to the top stimulus), while the other can be ignored. A value is assigned to every visual field, indicating whether the stimulus was located inside it. A value of 1 for a given visual field indicates that the stimulus is located entirely within that visual field, such as in Fig. 4a. However, the stimulus can be straddling two visual fields, such as in Fig. 4b. Consequently, the program attributes different values depending on the portion of the stimuli extent (light blue dashed lines) located in a visual field (the stimulus extent is here defined by its borders)

Using the visual fields previously defined and the location of the stimuli, the program assesses in which portion (frontal or lateral) of the hemifield (left or right) the stimuli fall in each frame. For each frame, if the stimulus(i) is located within a visual field, a value of 1 is attributed to that visual field (see the light blue dashed line in Fig. 4a). If the stimulus is straddling two visual fields, the proportion of the object located within each visual field is attributed to each one of them (see the light green dashed line in Fig. 4b). The output through which Visual Field Analysis provides eye use data varies depending on the location and number of stimuli. In this experiment, Visual Field Analysis computed eye use for one stimulus.

#### Error threshold setting

**Fig. 5:**
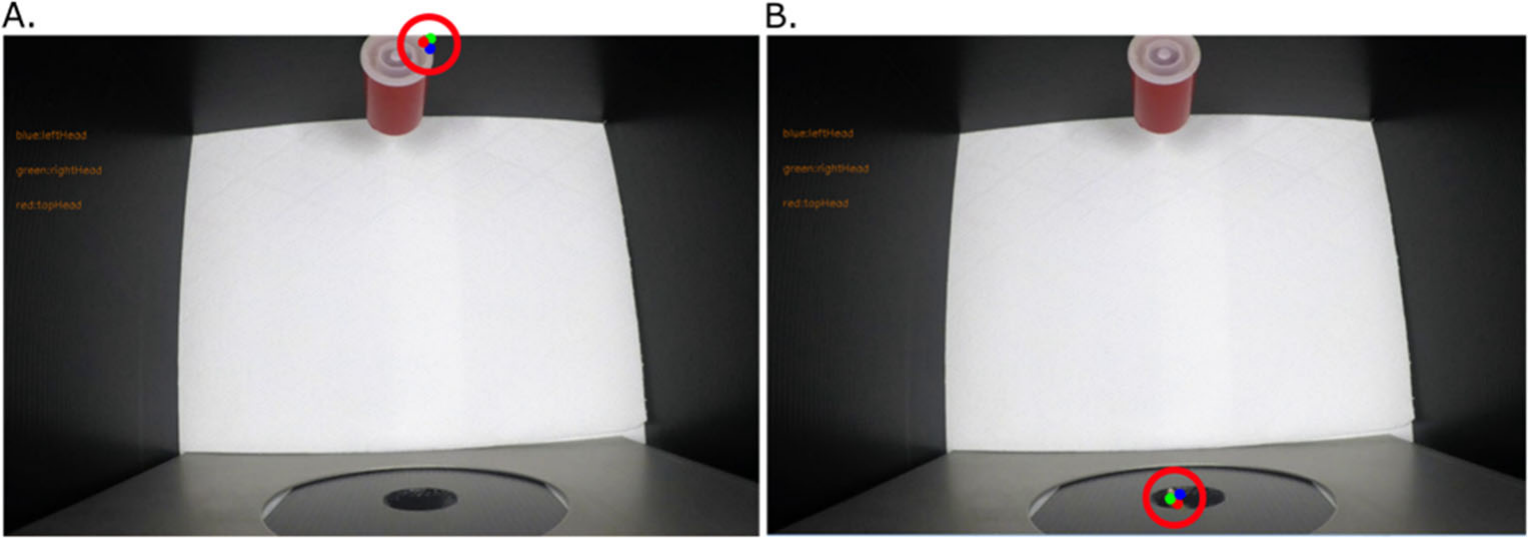
Two of the frames considered outliers in our example (from subject 5 of the current experiment), with a threshold of 3. The red circles on the images highlight the position of the labels. On image 5A, the chick has not placed its head inside the round opening yet, but DeepLabCut incorrectly placed the ‘leftHead’ (blue dot), ‘topHead’ (green dot) and ‘rightHead’ (red dot) on an empty portion of the screen, close to the stimulus. In image 5B, the chick started to insert its head in the round opening, but most of it is still invisible. DeepLabCut incorrectly located the ‘leftHead’, ‘topHead’ and ‘rightHead’ labels on the animal’s beak.

As a strategy to identify and exclude frames inaccurately tracked by DeepLabCut, we implemented an error threshold approach. For each video, the experimenter can set an error threshold that specifies the acceptable range of distances between the three points tracked on the head of the animal. The error threshold is based on the average distance between each body part tracked by DeepLabCut and excludes frames that are too far from the average distance. As an example, the average distance between the ‘leftHead’ and ‘rightHead’ points was 58 pixels for subject 5 of the current experiment. For this animal, we chose a threshold of 3. This threshold corresponds to the number of standard deviations above which a frame is considered an outlier; thus, the absolute value of the threshold in terms of pixels is variable. With a threshold set at 3, 1.86% of the frames were preliminarily labelled as outliers. These frames were then manually inspected to address the accuracy of this process and could be removed from our analysis if visual observation confirmed them to be outliers (Fig. 5). The threshold used for each subject of the current experiment and the percentage of frames manually checked and excluded from the analyses at these different steps are reported in Table 1.

**Table 1:** Table showing the tracking accuracy at the individual and at the group level. All the frames analysed by Visual Field Analysis were then manually coded, and the ‘frames relabelled’ column reports the number of frames for which a discrepancy emerged between manual and automated coding. The ‘frame verified’ column reports the tracking accuracy, which corresponds to the number of frames correctly tracked (‘frames verified’ minus ‘frames relabelled’) over the total number of frames (‘frames verified’). The two types of coding (manual and automatic) were compared using Spearman’s correlation tests, both at the individual level and for the whole sample of *N* = 10 chicks. This was done separately for each visual field (frontal left and right, lateral left and right). Results of these correlations are reported in the last eight columns

subjects ID	error treshold chosen	frames removed by VFA [%]	frames verified	frames relabelled	tracking accuaracy [%]	corr. frontal left		corr. frontal right		corr. lateral left		corr. lateral right	
						pearson’s r	p.value	pearson’s r	p.value	pearson’s r	p.value	pearson’s r	p.value
1	3	0	898	14	1.56	0.99	<0.001	0.97	<0.001	1.00	<0.001	0.99	<0.001
2	4	0	749	27	3.60	1	<0.001	0.97	<0.001	1.00	<0.001	0.97	<0.001
3	3	0	749	12	1.60	0.99	<0.001	1	<0.001	0.99	<0.001	1	<0.001
4	6	0	749	35	4.47	0.99	<0.001	0.89	<0.001	1	<0.001	1	<0.001
5	4	0	749	3	0.40	0.99	<0.001	0.99	<0.001	1	<0.001	1	<0.001
6	3	0	749	1	0.13	0.77	<0.001	1	<0.001	0.99	<0.001	1	<0.001
7	5	0	749	50	6.68	0.90	<0.001	0.91	<0.001	0.97	<0.001	1	<0.001
8	3	0	749	0	0	1	<0.001	1	<0.001	1	<0.001	1	<0.001
9	3	0.27	749	259	34.58	0.92	<0.001	0.63	<0.001	0.97	<0.001	0.82	<0.001
10	4	0	749	0	0	1	<0.001	1	<0.001	1	<0.001	1	<0.001
over all			7639	401	0	0.98	<0.001	0.96	<0.001	0.99	<0.001	0.98	<0.001

To help users choose an appropriate threshold and check the program’s accuracy, Visual Field Analysis has a built-in function to visualize frames. It is possible to visualize the frames removed from the analysis given the chosen error threshold, in addition to a set of randomly selected frames which are provided by the program to assess its accuracy.

#### Manual coding of the data

To assess the reliability of the eye use data provided by Visual Field Analysis, we manually checked all the frames analysed by Visual Field Analyses (7639 frames in total). The frames were checked and saved using a built-in function provided by Visual Field Analysis. Each frame was inspected by two independent coders, each of whom coded all the frames independently from the other. Then, the two experimenters compared their output, re-inspected all the frames for which a different coding was assigned and agreed on a final labelling of these frames.

The manual scores were attributed using the same score attribution method and the same visual field subdivisions as Visual Field Analysis. On each frame, the coders superimposed a transparent sheet, on which five lines originating from a central point represented the subdivisions of the chick visual field (see Fig. 3). For each frame, if the visual field delimitation lines of the translucent sheet used for manual scoring overlapped perfectly with the lines used by Visual Field Analysis for the attribution of the scoring (as visible in Fig. 4), the original automated scoring was considered accurate. In this case, the same original scoring provided by Visual Field Analysis was also reported for manual scoring. If there was not a perfect overlap, the original scoring was considered inaccurate and the frame was ‘relabelled’, providing new values for manual scoring. In this case, the same criteria as described above were followed: if the stimulus fell entirely within a visual field, a value of 1 was attributed to it. If the stimulus straddled two visual fields, the proportion of the stimulus located within each visual field was attributed to each one of them (e.g., 0.75 and 0.25).

### Statistical analyses

The scores obtained for each visual field (frontal left, frontal right, lateral left and lateral right) were compared between scoring methods (Visual Field Analysis vs manual coding) using correlation tests (Pearson’s test) overall and per individual. An estimation of the number of frames needed to achieve a significant power for the correlation analysis was run prior to the study, assuming an *r* value of 0.9 (given the high accuracy of the tracking methodologies). According to this estimation, it would be sufficient to code only seven frames to achieve a statistically significant correlation. However, we deemed this to be insufficient to provide a reliable validation of our software. Thus, in order to provide the most precise estimation possible of the accuracy of our method, we decided to code all the frames available, greatly surpassing the minimum number required to achieve sufficient statistical power. As some videos were better tracked by DeepLabCut than others, we report the reliability of our program in relation to the tracking accuracy (measured as the percentage of frames that received identical scoring in the manual and automated scoring). The statistical tests were performed using RStudio version 4.0.2 (RStudio Team, 2020).

## Results

### Eye use data reliability

The Pearson’s correlation tests revealed an almost perfect, and highly significant, correlation between scoring methods, both when the data for the whole sample were taken into consideration and when the analysis was run at the single subject level. This was true for each visual field (see Table 1 for statistics).

The number of frames for which the manual scoring of the human coder was discrepant from that assigned by Visual Field Analysis (relabelled frames) is detailed for each subject in Table 1. No discrepancy between the manual coding and the automated coding was found for subjects 8 and 10. Thus, for these subjects, no frames were relabelled, and a perfect correlation was, of course, found between manual and automated scoring. One should note that the reliability of our application is directly dependent on the DeepLabCut tracking accuracy, which differs across conditions (different videos settings) and individuals (different behaviours). When the DeepLabCut tracking was 100% accurate, the output produced by Visual Field Analysis perfectly matched the manual scoring done by the human coders.

For the eight remaining subjects, the tracking accuracy (i.e., the percentage of frames that received identical scoring in the manual and automated scoring) fluctuated from 65.4 to 99.9%. Nonetheless, the reliability of the program for scoring eye use remained relatively high in all conditions (see Table 1 for statistics). Even when the tracking accuracy was at its lowest (65.4% for subject 9), the correlation between the coding provided by Visual Field Analysis and the manual coding remained strong for most visual fields (Pearson’s *r* ranging from 0.77 to 0.97), although it decreased in the frontal right visual field (Pearson’s *r* = 0.63).

## Discussion

Automated and reliable assessments of visual field use can support the investigation of behavioural lateralization. Our results show that Visual Field Analysis can be reliably used to automatically assess eye use behaviour in animals with laterally placed eyes, replacing manual coding methods. The comparison between the manual and the automated scoring revealed a nearly perfect correlation between manual score and Visual Field Analysis score.

With optimum tracking conditions, the results provided by the application can be reliable at 100% (i.e., identical results can be obtained as with manual scoring). Visual Field Analysis excludes from the analyses frames with a low degree of likelihood (i.e., frames that DeepLabCut considered as being unlikely to be well tracked), thus keeping only frames with a level of confidence of being well tracked above 95%. It also excludes frames where the distance between the DeepLabCut’s labels is higher than a given threshold. Note that the two measures are often correlated: when DeepLabCut tracking is imperfect, both the number of frames considered as unlikely to be well tracked and the number of frames considered as outliers with the threshold method are high. Therefore, most of the frames that could be wrongly tracked are excluded from the analysis. Moreover, using a built-in function of our application, the user can manually visualize a certain number of random frames of a video to check the program performance. We suggest visualizing at least 100 frames per individual with more than 90% of tracking accuracy in order to achieve a performance similar to what is described in the current study. If the tracking accuracy is lower than 90%, we suggest training DeepLabCut again using a new set of frames or a different labelling method.

Our application opens a new range of possibilities for laterality research. It can be adapted to different species, when tested in controlled setups, and provides fast and reliable data acquisition. To date, we have run preliminary tests of our application with honeybees and zebrafish (unpublished data), in addition to domestic chicks, revealing good tracking performance with all these species. From the technical point of view, therefore, Visual Field Analysis offers sufficient flexibility to be employed in any species, as long as the visual field dimensions are known and the position of the eyes can be reliably estimated when it is video-recorded from above. We thus consider this application as also highly suited to studies that compare the performance of different species, especially among animals with laterally placed eyes and relatively weak inter-hemispheric connections (such as many non-mammalian vertebrates). In animals with frontally placed eyes and stronger inter-hemispheric coupling, eye use studies are less straightforward to interpret and thus less common. However, this is more reflective of the biological constraints of these species than a specific limitation of the current software, which in principle could be adapted to a variety of vertebrate species. For instance, even in the case of animals with frontally placed eyes, Visual Field Analysis can be used to measure the total amount of time spent looking toward a stimulus, regardless of the visual field used. This method could hence address issues of subjective coding on human and non-human animal playback experiments in which the position of the head is scored.

One of the advantages of Visual Field Analysis is that it allows the accurate processing of vast amounts of data, which would normally be unfeasible to process with manual coding methods. For instance, this kind of data can be easily gathered in cross-sectional studies in which the behaviour of the animals is continuously observed for protracted periods of time (rather than sampled at periodic intervals), as has been recently done in filial imprinting studies in chicks (Lemaire et al., 2021). Moreover, longitudinal studies investigating the ontogenesis and development of the same individuals over extended periods of their life span could also benefit from the ability to effortlessly process large quantities of data, as allowed by Visual Field Analysis. Note that a large amount of data can be processed even for short-duration tasks by recording the animal behaviour with high frame rates. This is particularly relevant to species moving their heads at a very high speed such as birds (Kress et al., 2015) and insects (Boeddeker et al., 2010; Hateren & Schilstra, 1999). The use of our application to encode visual field data could be thus particularly beneficial for these kinds of research.

Moreover, Visual Field Analysis not only assesses simple preferential eye use (whether the left or right eye is preferred); it can also investigate the use of sub-regions within each hemifield (frontal visual field vs lateral visual field), allowing the analysis of eye use behaviour at a fine level, which is very time-consuming if done manually as performed by Lemaire and collaborators (2019) in King Penguins. Alongside measuring eye use, the application allows the recording of other relevant behavioural measurements, such as the activity level of an animal’s head, while keeping track of its positions in different areas of a testing environment (for this last function, the performance of Visual Field Analysis was already validated in a previous study, showing once again very high correlation with the measurements obtained by traditional manual coding methods; Santolin et al., 2020). In addition, Visual Field Analysis can extract data on the movements of the heads of the animals, quantifying the amount of motion of this body part. This can be informative about changes in eye use and head saccades, a behaviour present in birds which is correlated with arousal (Golüke et al., 2019; Kjrsgaard et al., 2008). These other behavioural measurements provide additional information that can be analysed in relation to eye use behaviour or independently from it, allowing for richer behavioural assessments and more flexible use in different experimental designs.

However, our data acquisition technique is entirely dependent on tracking accuracy: a key component most tracking software still struggles with. To provide optimal results, in most tracking software the video recording quality has to be pretested and adjusted, while the experimental settings must be similar throughout the whole data acquisition phase. By using deep learning techniques, DeepLabCut started to overcome those limitations. For instance, DeepLabCut can be trained to recognize animal body parts in a wide range of scenarios as long as the video recordings are of sufficient quality. Moreover, DeepLabCut even allows the tracking of multiple animals at the same time (Lauer et al., 2021). In its current version, however, Visual Field Analysis allows the tracking of only one animal at a time, within an orthogonal arena. Moreover, the current version of this application provides only limited flexibility in the shape of the experimental arena and in the placement of the stimuli within it (see Josserand & Lemaire, 2020 for further details). Since this represents potentially the greatest limitation of this software, we are actively working to update Visual Field Analysis so that it can be used in a greater variety of experimental designs, both in terms of stimuli placement and in the shape of the experimental arena.

Given the numerous practical advantages offered by automated behaviour tracking methods compared to manual ones, we believe that automated methods should be chosen to ensure reproducible data analysis. Visual Field Analysis offers an important resource for research on behavioural lateralization, enabling the collection and analysis of a richer set of data, in a less time-consuming and more unbiased way.
